# A Review of Recent Advances in Fractional-Order Sensing and Filtering Techniques

**DOI:** 10.3390/s21175920

**Published:** 2021-09-02

**Authors:** Cristina I. Muresan, Isabela R. Birs, Eva H. Dulf, Dana Copot, Liviu Miclea

**Affiliations:** 1 Automation Department, Technical University of Cluj-Napoca, 400114 Cluj-Napoca, Romania; Cristina.Muresan@aut.utcluj.ro (C.I.M.); Eva.Dulf@aut.utcluj.ro (E.H.D.); Liviu.Miclea@aut.utcluj.ro (L.M.); 2 Dynamical Systems and Control Research Group, Ghent University, 9052 Ghent, Belgium; Dana.Copot@ugent.be; 3 Core Lab EEDT, Flanders Make Consortium, 9052 Ghent, Belgium

**Keywords:** fractional calculus, fractional-order filters, fractional-order sensors, fractional-order analog filters, fractional-order digital filters, fractional-order applications

## Abstract

The present manuscript aims at raising awareness of the endless possibilities of fractional calculus applied not only to system identification and control engineering, but also into sensing and filtering domains. The creation of the *fractance* device has enabled the physical realization of a new array of sensors capable of gathering more information. The same fractional-order electronic component has led to the possibility of exploring analog filtering techniques from a practical perspective, enlarging the horizon to a wider frequency range, with increased robustness to component variation, stability and noise reduction. Furthermore, fractional-order digital filters have developed to provide an alternative solution to higher-order integer-order filters, with increased design flexibility and better performance. The present study is a comprehensive review of the latest advances in fractional-order sensors and filters, with a focus on design methodologies and their real-life applicability reported in the last decade. The potential enhancements brought by the use of fractional calculus have been exploited as well in sensing and filtering techniques. Several extensions of the classical sensing and filtering methods have been proposed to date. The basics of fractional-order filters are reviewed, with a focus on the popular fractional-order Kalman filter, as well as those related to sensing. A detailed presentation of fractional-order filters is included in applications such as data transmission and networking, electrical and chemical engineering, biomedicine and various industrial fields.

## 1. Introduction

The number of fractional calculus applications has seen a rapid growth over the last decade. Fractional calculus can be easily defined as a generalization of integer-order calculus with the order of the differintegral operators as fractional. Its versatility in modeling and control theory has received a lot of attention recently, although it is still a concept insufficiently understood. This limits the wide acceptance of fractional calculus in industrial use. Fractional calculus has been regarded as a much better way to cover the dynamics of certain type of phenomena, such as anomalous diffusive characteristics [1], viscoelasticity [2], epidemic spreading [3], etc. At the same time, fractional calculus in controller design has increased their flexibility and robustness [4,5]. Review papers dealing with the use of fractional calculus in control engineering have been published recently, such as [3,6,7,8,9].

However, apart from fractional-order models and controllers, the theoretical aspects of fractional calculus have been extended to cover adjacent areas of research, namely sensing and filtering. This has somewhat evolved as a logical step, since actual processes are better modeled using fractional-order systems [10]. At the same time, state estimation is crucial in designing fractional-order controllers [11]. Thus, for a robust state estimation and an efficient noise elimination in fractional-order systems, extensions to a fractional-order of the popular integer-order estimators have been proposed.

It has been widely proven that complex systems can be accurately described by power-law series [12]. For the case of electronic devices, the behavior is given by the sum of various independent actions of the charge carriers, exhibiting the normal distribution. Unknown interactions in the electronic device leads to the second moment of distribution that fails to converge. For the case of real-time sampling, the mean converges rapidly towards infinity, while the standard deviation fluctuates. These systems are best described by the Generalized Law of Large Numbers, resulting in power-law series behavior, with an added α-stable component, proving the presence of fractional-order dynamics in any complex system [13,14]. Hence, fractional-order sensors can provide a powerful tool in acquiring more accurate data regarding the surrounding environment, as will be shown in the present study.

Filters are one of the key elements in the signal processing field. Many filtering techniques have been developed throughout the years for noise reduction, signal modulation, demodulation, amplification, etc. Filters can be analog, consisting of electronic circuits that process the analog signal, or digital, consisting of mathematical filters that process the analog signal after its discretization. The popular field of fractional calculus has also infiltrated into filter design, for both analog and digital cases. For the case of analog filters, the creation of the *fractance* device, integrating fractional-order dynamics into electronic components such as the fractional-order capacitor has been the starting point of fractional-order analog filters. Fractional-order electronic components are used to create filters that have a larger frequency range and a better response than integer-order filters [15]. However, due to the limitations present in fractional-order physical hardware, there are only a few studies covering the physical realization of fractional-order analog filters, which will be described later in the manuscript.

Fractional-order digital filters are more abundant in the specialized literature since it is much easier to implement a mathematical relationship on a suitable processor than creating its hardware counterpart. Most fractional-order filters cover the extension of the widely popular Kalman filter into the fractional calculus domain. For integer-order linear systems, Kalman filters (KF) are a convenient way to handle state estimation and Gaussian noise [16]. For the approach to work, complete prior knowledge of system models and noise parameters is necessary. The major drawback here refers to the difficulty of knowing noise parameters in advance [17]. To improve the performance of the standard Kalman filter, several extensions and modifications have been proposed, including the extended Kalman filters (EKF) [18,19]; the unscented Kalman filters (UKF) [20]; particle filters (PF) [21] or cubature Kalman filters (CKF) [22] to name just a few. Apart from the wide variety of Kalman-based filters, there are also the Butterworth-type fractional-order digital filters and fractional-order delay filters that have seen an increased popularity in the last decade.

The applicability of digital fractional-order filters spans on a manifold of domains from data transmission and networking applications [23,24,25], electrical vehicle manufacturing (through the determination of state of charge in lithium-ion batteries [26], aerial vehicle orientation using fractional-order filtering of yaw, pitch and roll signals [27], air-quality assessments through pollution and humidity factors [28], civil engineering targeting the measurement and data processing of various characteristics of buildings such as stiffness and damping [29], different biomedical processes, image processing and many more. Most of the existing implementations of fractional-order filters are in the fields of data transmission and battery estimation, as will be shown in a dedicated section that highlights the benefits of fractional-order filters in real-life applications.

This paper covers a review of fractional-order sensing, as well as estimation methods using fractional-order filters. The focus is on the most recent findings in this domain, covering the past decade. The paper is structured as follows. The next section briefly goes over the definitions of fractional-order operators to serve as a starting point for understanding the basis of fractional-order dynamics in both sensors and filters. Fractional-order sensors are covered in the following section. Due to the limited research in this topic, only a few papers and their main results are covered here. The main topic of this survey paper is detailed in a subsequent section that deals with the most recent advances and findings on fractional-order filters. The section is divided into two main parts covering analog and digital filters including here, but not limited to, fractional-order Butterworth filters, fractional-order delay filters and variations of the popular Kalman filter. The last section details some recent publications on the applications of fractional-order filters. The main application areas covered are data transmission and networking, battery state-of-charge estimation, biomedical engineering, aerodynamics, vehicle tracking, environmental issues, etc. Finally, a discussion section presents an overview of the findings related to fractional-order sensing and filtering together with current trends and research directions.

## 2. An Insight into Fractional-Order Calculus

This section focuses on briefly presenting the mathematical tools on which fractional-order sensing and filtering are based.

The fractional operator is denoted by ${}_{a}D_{t}^{\alpha }f\left(t\right)$, with t,a∈R, where (t>a) are the upper and lower limits of the *differintegral*, representing a generalization of integral and derivative operations to any arbitrary order, α∈R.

The most widely used definitions of ${}_{a}D_{t}^{\alpha }f\left(t\right)$ have been introduced by Riemann–Liouville (RL), Grünwald–Letnikov (GL) and Caputo (C) [30].

Fractional-order control strategies are mainly developed based on the Riemann–Liouville definition given as

(1) $${}_{a}^{RL}D_{t}^{\alpha }f\left(t\right)=\frac{1}{\Gamma (n-\alpha )}\frac{d^{n}}{dt^{n}}\int_{a}^{t}\frac{f(\tau )}{{\left(t-\tau \right)}^{\alpha -n+1}}d\tau ,$$

where n−1<α<n, with *n* being the smallest integer greater than α and Γ(n−α) is the Euler gamma function [31]. The upper and lower bounds, *t* and *a*, need to be established in the case of the RL definition.

Another popular representation of the fractional differintegral operation is the Caputo fractional derivative, introduced in 1967 by Michele Caputo [32]. The advantage of this definition is that the fractional-order initial conditions do not have to be defined as in the RL case.

(2) $${}_{a}^{C}D_{t}^{\alpha }f\left(t\right)=\frac{1}{\Gamma (n-\alpha )}\int_{a}^{t}\frac{f^{n}\left(\tau \right)}{{\left(t-\tau \right)}^{\alpha -n+1}}d\tau ,$$

with n−1<α<n and α>0.

Grünwald–Letnikov (GL) defines ${}_{a}D_{t}^{\alpha }f\left(t\right)$ as

(3) $${}_{a}^{GL}D_{t}^{\alpha }f\left(t\right)=\lim_{h\to 0}h^{-\alpha }\sum_{m=0}^{\left[\frac{t-a}{h}\right]}{\left(-1\right)}^{m}\left(\begin{array}{c} \alpha \\ m \end{array}\right)f\left(t-mh\right),$$

where α∈R and α>0.

A numeric approximation of the GL definition from Equation (4) has been proposed by [33] as

(4) $$\begin{array}{c} \begin{array}{cc} {}_{a}^{GL}D_{t}^{\alpha }f\left(t\right) & =T^{-\alpha }\sum_{m=0}^{M(t)}{\left(-1\right)}^{m}\left(\begin{array}{c} \alpha \\ m \end{array}\right)f\left(t-mT\right)\\ \; & =T^{-\alpha }\sum_{m=0}^{M(t)}c_{m}^{\left(\alpha \right)}\left(\begin{array}{c} \alpha \\ m \end{array}\right)f\left(t-mT\right) \end{array} \end{array}$$

where *T* denotes the sampling time and *L* is a memory length chosen to satisfy

(5) $$\begin{array}{c} \begin{array}{cc} L & \geq \frac{1}{\delta_{0}^{2}\Gamma \left(\alpha \right)}\\ \delta_{0} & =\frac{{|}_{a}^{GL}D_{t}^{\alpha }f\left(t\right){-}_{t-L}^{GL}D_{t}^{\alpha }f\left(t\right)|}{P}\\ P & =\max_{\left[0,\infty \right]}\left|f(t)\right| \end{array} \end{array}$$

M(t) is the minimum between t/h and L/h, while $c_{m}^{\left(\alpha \right)}$ are binomial coefficients defined as

(6) $$c_{m}^{\left(\alpha \right)}=\left(1-\frac{1+\alpha }{m}\right)c_{m-1}^{\left(\alpha \right)}$$

with $c_{0}^{\left(\alpha \right)}=1$.

Another important piece in fractional calculus theory is the Mittag–Leffler function

(7) $$E_{\alpha }\left(t\right)=\sum_{m=0}^{\infty }\frac{t^{m}}{\Gamma (\alpha m+1)}$$

that connects the pure exponential and power-law behavior, characterizing both integer and fractional-order phenomena [34], resulting

(8) $$L\left\{E_{\alpha }\left(\pm at^{\alpha }\right)\right\}=\frac{s^{\alpha -1}}{s^{\alpha }\mp a}.$$

A more recent fractional-order definition has been proposed by Coimbra in 2003 [35] known throughout the specialized literature as the Variable-Order (VO) differential operator

(9) $$\begin{array}{c} \begin{array}{cc} {}_{}^{VO}D_{t}^{\alpha (t)}f\left(t\right) & =\frac{1}{\Gamma (n-\alpha (t))}\int_{0}^{t}{\left(t-\tau \right)}^{-\alpha (\tau )}\frac{df(\tau )}{d\tau }+\frac{\left(f\left(0^{+}\right)-f\left(0^{-}\right)\right)t^{-\alpha \tau }}{\Gamma (1-\alpha (t))}. \end{array} \end{array}$$

A simpler alternative to the highly popular RL definition has been offered by Jumarie in 2006 [36] as

(10) $$\begin{array}{c} \begin{array}{cc} {}_{}^{J}D^{\alpha }f\left(t\right) & =\frac{1}{\Gamma (n-\alpha )}\frac{d^{n}t}{dt^{n}}\int_{0}^{t}\frac{f(\tau )-f(0)}{{\left(t-\tau \right)}^{\alpha +1-n}}d\tau . \end{array} \end{array}$$

The VO, RL and Jumarie approaches are recognized as a powerful mathematical tool for modeling viscoelastic phenomena such as deformation, viscoelastic fluid flows and interactions, anomalous diffusion in porous media and various biomedical processes. It has been proven that integer-order calculus cannot accurately describe complex diffusion processes due to the limitations brought by the constant order, but a variable order of differentiation is a natural solution to determine diffusion patterns [36,37]. The fractional VO definitions does not hold the common properties of a derivative and are considered extremely difficult to compute analytically, most often being approximated with numerical approaches [35].

A major advantage associated with the RL definition is that f(t) does not have to be continuous at the origin, nor differentiable. Furthermore, since Jumarie is a modified RL derivative, f(t) does not have to be differentiable and the derivative of a constant is zero. Another advantage of both Jumarie and RL is that there is no singularity at the origin for all functions, such as ML. Modeling physical phenomena with the RL definition is a tedious process since the RL derivative of a constant is different than zero. Furthermore, a constant f(t) at the origin leads to a singularity in its fractional derivative, reducing its field of applications [38]. A drawback associated with Jumarie is that if f(t) is discontinuous at the origin its fractional-order derivative does not exist [39].

The main advantage brought by the Caputo derivative is that it allows the specification of initial and boundary conditions in a traditional manner and the derivative of a constant is also zero. This is the reason Caputo is considered the most appropriate definition for modeling real-world problems [40]. However, the Caputo derivative requires the computation of the derivative of f(t) to compute its fractional-order derivative. There is also the limitation that Caputo defines the differintegral only for differentiable functions and functions that do not have a first-order derivative could have fractional derivatives of all orders [41].

## 3. Fractional-Order Sensing

The most prevalent advantages of applied fractional-order differintegrals are widely proven in process control as shown by multiple comprehensive review papers on the fractional-order PID controller [6,42,43] and complex system modeling in biomedical processes [2] through complex anomalous diffusion [9]. However, during recent years different fields such as fractional-order circuits had undergone the possible integration of fractional operators. Fractional-order sensors are a novel topic of applied fractional calculus and its full potential is yet to be grasped by the scientific community.

The aim of this section is to provide some basic operating principles of fractional-order dynamics in real-world sensors and current advances in sensing using this approach.

A fractional-order sensor can be defined as an electronic device that incorporates fractional-order devices and their evident dynamics in its construction. The first element built such that its impedance followed a power-law frequency of fractional-order is known in the specialized literature as the *fractance* device. Power-law dynamics have been successfully implemented in electronic devices using impedance spectroscopy, encapsulating fractional calculus with the help of “fractional capacitors”, as shown in the extensive review from [44]. The design, manufacturing and testing of various fractional-order device prototypes have been presented through different approaches. An in-depth review regarding construction possibilities, materials and techniques has been published in [45]. The circuit and system design theory present behind these fractional-order components is extensive and is out of scope of the present paper.

Furthermore, the review will discuss only studies that integrated fractional-order components to build electronic devices with sensing capabilities. The focus is to provide the reader with an idea towards the real-life applicability of fractional-order sensors, without going into too much detail regarding their physical realization.

A novel architecture of a fractional-order sensing device is explored in [46] based on the behavior of the popular accelerometer combined with fractional calculus. The construction of the sensor is presented as a modular device with N stages, each consisting of performing an analogy with the mass-spring-damper device. The paper shows that properly selecting the properties of the mass-spring-damper construction leads to fractional dynamics in the resulting circuit. Two possibilities of choosing the optimal parameters are presented based on recursive formulas and particle swarm optimization. The presented fractional-order sensor is presented solely in the conceptual stage. However, the authors state that the device can be physically realized using micro electro-mechanical components, but the advantages of using it in an industrial and/or commercial setting still need to be determined.

A fractional-order sensor for measuring the quality of milk is proposed in [47]. The authors develop a nanostructured aluminum oxide (Al${}_{2}$O${}_{3}$) constant phase impedance sensor (CPI) by depositing porous oxide film on the double-sided metal plated substrate. Porous film was fabricated by sol-gel technique and electrochemical anodization. When the sensor was immersed in the ionic medium of milk samples, it showed constant phase behavior over a certain frequency range, but the phase angle changes due to change in the type of the milk samples. Experiments were conducted with the CPI sensor for different adulterated milk samples and the sensor fabricated with anodized oxide film show better performance for analyzing the quality of pure and adulterated milk.

A new stick type sensor is proposed in [48] to analyze the quality of drinking water. The sensor exhibits constant phase behavior which clearly indicates a fractional-order impedance. The research shows that the variation of the ionic impurities in water lead to a variation of the parameters of the sensor, the constant phase angle and the fractional exponent. The preliminary results showed that the sensor could be a viable choice for the development of a ball pen type probe for testing drinking water including its adulteration with impure water.

Fractional-order circuits are used in [49] to develop a voltametric sensor used for taste measurements of black tea liquor. The work is the first proposal of a fractional sensing device in tasting applications and presents an experimental electronic tongue built using fractional-order components. The authors start from the premise that the response of a voltametric sensor is best modeled using fractional-order mathematical models and develop a fractional-order circuit that registers the current response. A fractional-order equivalent circuit is used to obtain an accurate model of the system which is further decomposed into different elements with optimally selected parameters. The paper uses a direct approach, with an additional novelty in completely avoiding the measurement of a fractional-order impedance circuit.

The study published by [50] focuses on creating a new inductive transducer by combining an impedance converter with a fractional-order element realized as an RC ladder network. The paper presents both the construction details of the real-life sensing device together with a working example consisting of the measurement of an input displacement. The results obtained with the proposed fractional-order sensing device are compared with different sensing approaches, proving the efficacy of fractional-order circuits in better measuring physical phenomena. Furthermore, it is shown that incorporating fractional-order devices in the electronic circuit improves the sensitivity and compensates for the transducer’s nonlinearity with possible applications in a wide variety of measurements such as force, pressure, etc.

## 4. Fractional-Order Filters

### 4.1. Analog Filters

Fractional-order circuits such as the previously mentioned *fractance* are employed to create various analog filters with fractional-order components. The main advantages of this type of filters are speed and operating conditions on a larger frequency range, with the fractional element that provides a greater flexibility in shaping the frequency response. Among the disadvantages, there is the need for physical space for the hardware components, design and manufacturing of the electronic circuit, and inability to modify it on the go. Furthermore, from the fractional-order filter perspective, there are also disadvantages related to the scarcity of fractional-order electronic components. However, there are several recent studies in the specialized literature that use fractional-order dynamics in analog filters. Sotner et al. published in [15] an interesting comparison between an integer and a fractional-order filter built using an integer-order capacitor and a fractional-order capacitor, respectively. There are several differences outlined in the study between the responses obtained with the two approaches, but the general conclusion is that the fractional-order filter is more versatile.

One of the most prevalent analog fractional-order filters is the Butterworth filter with various alterations. The concept of analog Butterworth filter has been extended for fractional-order systems using the complex ω plane instead of the Laplace *s* transform in [51]. The paper presents an in-depth mathematical analysis related to stability together with simulation results and a set of guidelines for the physical realization of the filter. The simulation results prove that the designed fractional-order filter is superior to integer-order Butterworth filters through various comparisons on different frequency ranges and filter orders. Furthermore, it is clearly shown that the fractional filter design technique can meet exact frequency domain specifications, as opposed to integer-order filter design.

A differential voltage current converter and two fractance devices are used in [52] to create a real-life representation of a fractional-order Butterworth low-pass filter. The paper tackles stability of the filter using Monte Carlo analysis and investigates its behavior using different circuit parameters. The fractance device is also used in [53] to design a fractional-order low-pass Butterworth analog filter. The parameters of the filter are determined using evolutionary optimization techniques consisting of the Artificial Bee Colony Algorithm. The applicability of the proposed method is validated experimentally for real-time processes, showing that fractional-order filters can successfully replace higher-order integer systems. Another fractional-order low-pass filter with Butterworth characteristics is proposed in [54]. The study focuses on presenting novel topologies of complementary fractional-order filters based on approximation algorithms such as Continued Fraction Expansion and Oustaloup approximation. The paper also presents the circuit implementation of the proposed filters using MOS transistors and evaluates the behavior of the filters for fractional-order of α=0.3,0.5,0.7.

Reference [55] presents the design of a fractional-order analog pseudo-differential frequency filter with order 2+α, where α∈(01). The resulting filter is a low-pass Butterworth that employs a minimum number of passive components and current conveyors as active elements. The filter is validated from both simulation and experimental points of view through a custom PCB prototype. The study proves that the designed fractional-order filter has a high common mode rejection ratio and low total harmonic distortion. Both low-pass and high-pass Butterworth filters are introduced by [56], this time with order 1+α, where α∈(01). The filter coefficients are computed based on a tradeoff between stability margin and magnitude error. The study uses a field-programmable analog array (FPAA) to experimentally validate the fractional-order filter, proving robustness, reduced sensitivity to parameter variations and reduced errors for magnitude, passband and stopband responses.

In [57], a different fractional-order filter is built using Current Feedback Operational Amplifiers as an active element. The design procedure is exemplified for a 1+α, α∈(01), order low-pass filter using experimental results. One of the advantages of the study is the usage of commercially available components to realize the fractional-order filter. Study [58] presents the realization of a 2α fractional-order band-pass filter using Operational Transconductance Amplifiers as active elements. The proposed methodology results in a tunable filter, featuring an electronic tuning possibility by changing the bias current. The filter is successfully validated using parameter uncertainties, stability and the effect of transistor mismatch. Current Feedback Operational Amplifiers are also used in [59,60] to realize low-pass, high-pass and band-pass filters of fractional orders. The papers use fractional-order resistor and capacitor banks to illustrate the physical realization of the filter.

The authors of [61] present a detailed study on the design, analysis and physical realization of analog low-pass, high-pass and band-pass fractional-order filters. The paper proposes a design strategy that uses *s* domain transfer functions, as opposed to similar works that use the ω plane (such as [51,53]). The implementation possibilities of the obtained fractional-order filters are versatile, featuring fractional Tow-Thomas capacitors, FPAAs, Single Amplifier Biquads or Frequency Dependent Negative Resistors.

Universal single input multi-output fractional-order filters are designed in [62] using DVCC+ block, capacitors and resistors. Unlike the previously presented works in this subsection, the paper exemplifies all types of filters: low-pass, band-pass, high-pass, all-pass and notch. The effects of the fractional-order term are analyzed for α∈(0.11.2) with respect to noise rejection, cutoff frequency, gain and phase. A comparison with already reported results shows that the proposed methodology yields a similar performance.

Another low-pass fractional-order filter is presented in [63]. The novelty of the study is the usage of a fractional-order capacitor built from multi-walled carbon nanotubes. The paper shows the fractional-order dynamics of the electronic element and the realization of the filter with a biasing current source. The study is backed by experimental results.

A novel category of fractional-order filters has been recently introduced by [64]. The proposed methodologies use curve fitting methods to completely eliminate the usage of complex fractional-order Laplace operators. The main scientific contribution of the work consists of providing a class of fractional-order filters that can be obtained using available integer-order elements, without compromising the fractional-order dynamics of the resulting devices.

The same authors also proposed double exponent fractional-order filters in [65]. The proposed fractional-order filters can be designed as both low-pass and high-pass filters with the transfer function similar to a second-order filter, but with arbitrary fractional orders α and β. The filters are approximated to their integer-order equivalent using Pade approximation and curve fitting tools. The paper also presents the physical realization of the proposed filters using RC networks and electronically tunable Operational Transconductance Amplifiers.

The study published by [66] raises awareness of the possible benefits of fractional-order analog filters in a wide range of domains such as video signal processing, sonar receivers, radar devices, biomedical analog signal processing, etc. The common point of the previously mentioned applicability of fractional filters is the necessity of a linear phase response. For this purpose, ref. [66] proposes a methodology for fractional-order analog filter design using Optimal Bessel Filters. It is shown that the obtained filter reduces overshoot, eliminates ringing with minimal phase distortion and provides a better transient response than an integer-order filter.

### 4.2. Digital Filters

From the fractional-order perspective, digital filters are much more common than analog ones since they completely eliminate the need for a separate physical device. Digital filters are generally more versatile and can be easily implemented as a mathematical formula on any system with a processor. There are no additional costs such as hardware equipment and no external influences (temperature, humidity, etc.) on the long-term functioning of the filters. However, digital filters are slower than analog ones, introduce additional latency and they require previously acquired and/or processed data. A disadvantage encountered in digital integer-order filters is a more limited frequency range when compared to integer-order analog filters. However, it has been shown that using fractional-order digital filters can overcome the frequency range limitations of integer-order ones, obtaining a reduced quadratic error between the desired frequency response and the obtained filter [67,68].

The popular analog fractional-order Butterworth filter has multiple digital implementations proposed by [68,69,70,71,72]. Analog-to-digital transformations of a fractional-order Butterworth filter are used in [69] using the infinite impulse response and Al-Alaoui operator, followed by global search constrained evolutionary algorithms to determine the parameters of the filter. Infinite impulse response is also used in [70] to design direct digital fractional-order Butterworth filters through optimization routines. A digital filter for image sharpening applications is proposed by [71] starting from the integer-order Butterworth representation. Mathematical tools such as discrete cosine, sine and Fourier transforms together with the Prony and Farrow methods are employed. The results prove the effectiveness of the proposed filtering strategy on real-life image sharpening use cases.

A class of purely digital filters are the fractional-order delay filters. The scope of such a filter is to delay the signal with a fractional of the sampling time. The authors of [73] state that two main frequency domain specifications should be met to obtain the fractional delay filter characteristic: the magnitude frequency response should have an all-pass characteristic, while the phase plot must have a slope that is fixed and linear throughout the entire bandwidth. The specialized literature reports two main design strategies where the filter coefficients are computed using mathematical interpolation formulas such as [74,75] or directly frequency domain optimization algorithms [76,77].

The majority of fractional-order filtering literature from the last decade focus on the extension of the well-known Kalman filter in the frequency domain resulting in three widely used fractional-order Kalman filters: the fractional-order Kalman filter (FKF), the fractional-order extended Kalman filter (FEKF) and the fractional-order unscented Kalman filter (FUKF).

State estimation is a tedious task when it comes to fractional-order systems. The complexity increases when dealing with nonlinear fractional-order systems, affected by delays, missing measurements and various types of noises. Additional problems need to be solved when the fractional-order system has a continuous-time form. Unlike integer-order systems, the state estimation of fractional-order systems requires a lot of historical data of input and output signals [78] due to the memory property of these particular systems. At the same time, for an accurate estimation, the measured signals must be filtered to remove noise [79]. As with the fractional-order models and controllers, researchers have demonstrated that better accuracy can be achieved when using a fractional-order state estimator. Thus, the fractional Kalman filter emerged [80]. Initially, these filters were used to estimate the states of simple discrete-time fractional-order systems. Later, extensions have been considered for delayed systems as well. The necessity of dealing with the state estimation of nonlinear fractional-order systems lead to the development of the extended Kalman filter and the unscented Kalman filter. Two major drawbacks of the fractional extended EKF have been identified and refer to the differentiability of the dynamic and measurement models and to the approximation of the nonlinearity by neglecting the higher-order terms in the Taylor series expansion. Several ideas to solve this issue have been proposed such as a statistically linearized method and cubature transform for state estimation in fractional nonlinear systems [81]. These has paved the way for several development of fractional-order cubature Kalman filters.

Dealing with continuous-time fractional-order systems represented a subsequent challenge, as the fractional-order system had to be differentiated. Discretization became an important research topic when designing a Kalman filter for continuous-time fractional-order systems [11]. Researchers have proposed several combinations between existing filters and discretization schemes, among which the Grünwald–Letnikov difference or the Tustin generation function and fractional-order average derivative are the most widely used. An additional challenge implies dealing with non-Gaussian noises in state estimation of fractional-order systems. Several studies have emerged, with solutions proposed for an accurate estimation of fractional-order systems states affected by Lévy noises and colored noises.

The fractional-order Kalman filter (FKF) and the fractional-order extended Kalman filter (FEKF) were the first ones developed to estimate the states and parameters of discrete fractional-order state models [80]. A general form of this FKF is presented in [82]. Additionally, state estimation for discrete-time fractional-order systems with delay is studied in [83,84]. A fractional Kalman filter-based multirate sensor fusion algorithm is presented in [85] to fuse the asynchronous measurements of the multirate sensors. The state is re-estimated whenever a delayed measurement occurs using a weighted fractional Kalman filter. A standard Kalman filtering method is then used to estimate the state estimation at the current time when the delayed measurement arrives.

To deal with nonlinear characteristics, the FEKF and the fractional-order unscented Kalman filter (FUKF) have been developed. Such a generalization of the FEKF to the case of uncertain observations is developed in [86]. The same authors also design an UKF where the scaled unscented transformation provides approximations of the first and second-order statistics of a nonlinear transformation of a random vector. Here, the nonlinear system is represented by a fractional-order discrete state-space system with uncertain observations, while independent Bernoulli random variables model the random interruptions in the observation process. A dual estimation algorithm is later designed for nonlinear fractional-order systems based on the fractional-order UKF [87]. The authors of [88] argue that the performance of the estimation result is affected by missing measurements and additive uncertainty in the gain. Since accurate and effective state estimation is essential for nonlinear fractional-order systems, a novel robust extended fractional Kalman filter (REFKF) is developed in [88]. The simulation results demonstrate that the nonlinear fractional-order system states can be accurately estimated even with missing measurements. Comparisons with the conventional FEKF show that the proposed method achieves better estimation performance. A robust state estimator for discrete-time nonlinear fractional-order systems is developed in [89]. The same issue regarding incomplete measurement data is tackled. A nonlinear fractional-order Kalman filter is developed to provide a more reliable and robust state estimation algorithm when both missing measurements and stochastic nonlinearities affect the system. Two numerical examples are used to validate the results.

The convergence of the FUKF is analyzed based on Lyapunov functions for nonlinear fractional-order systems, with the results indicating the divergence of the algorithm in the case of huge estimation errors. An adaptive noise covariance is suggested to overcome these huge estimation errors in [90] based on a fuzzy logic-based approach and a modified FUKF algorithm is proposed. The proposed algorithm is implemented and tested on a two electric pendulum system. The simulation results show that for the modified fuzzy logic FUKF algorithm produces accurate state estimation.

To deal with the drawbacks of the FEKF, several ideas have been proposed such as a statistically linearized method and cubature transform for state estimation in fractional nonlinear systems [81]. The approach is validated through numerous simulation results and its effectiveness is compared with the FEKF. A novel class of fractional interpolatory cubature Kalman filters (FICKFs) are designed in [91], as a generalization of the fractional cubature Kalman filter (FCKF) and the fractional unscented Kalman filter (FUKF). Based on interpolatory cubature rule, the FICKF algorithm achieves a custom degree of accuracy under accuracy under the Bayesian filtering framework. A robust FICKF algorithm is proposed by combining a traditional FICKF and an uncertainty estimator to estimate the states of a fractional-order uncertain nonlinear system. A hybrid version of the robust FICKF is also developed to ensure accurate estimation both in the presence and in the absence of uncertainty. To achieve this, the new algorithm has a switching mechanism between a FICKF and a robust FICKF. The validation of the algorithm is performed for state estimation of Malaria fractional nonlinear model with temporary immunity. The results show that the FICKFs with suitable free parameters lead to better accuracy compared with the existing filters with the same degree [91]. For nonlinear discrete-time fractional-order systems affected by colored noise, a similar FICKF is proposed in [92]. The authors propose a transformation of the system with colored noise into one with correlated process and measurement noises. Based on the extension of the measurements differencing method, new auxiliary outputs are introduced. The novel filtering algorithm is then applied to these new outputs. Simulation results on a fractional-order hyperchaotic Lorenz system targeting the cryptography in a communication system demonstrate the effectiveness of the proposed scheme [92].

Discretization becomes an important step when designing a Kalman filter for continuous-time fractional-order systems [11]. The Grünwald–Letnikov difference is used to design the FKF in [93]. An improvement of the state estimation accuracy using the concept of fractional-order average derivative was obtained for FKF in [94]. To overcome the effect caused by the colored measurement and process noises, the FKF in [95] is designed according to the Tustin generation function and fractional-order average derivative. In [11], FEKFs are designed using fractional-order average derivative and the Grünwald–Letnikov difference to handle state estimation for colored process noises and measurement noises, respectively. The idea of colored process noise is related to Kalman filtering and suggests that the system’s state changes over time, but without information on the cause or timing of the change which is modeled as a random process. Similarly, the FEKF in [79] is designed based on the fractional-order average derivative method and Tustin generating function for the nonlinear fractional system with uncorrelated and correlated noises. The results show that better estimation is obtained in this case compared to [78] or [94] where only the problem of uncorrelated noise is discussed. The Grünwald–Letnikov difference method is also used in [96], where two types of adaptive Kalman filters are developed. To deal with the nonlinearities, an adaptive extended Kalman filter is designed using the first-order Taylor expansion. Additionally, an adaptive cubature filter is developed based on the third-degree spherical-radial rule and the augmented vector method. Numerical examples are included to validate the effectiveness of the proposed adaptive Kalman filters with unknown parameters and fractional orders.

Hybrid fractional Kalman filters are also a suitable alternative for continuous-time fractional-order systems. In [97,98], the Grünwald–Letnikov difference and the fractional-order average derivative method are used to discretize a nonlinear continuous-time fractional-order system. To handle the nonlinearities, extended Kalman filter (EKF) and the unscented Kalman filter (UKF) are used. The EKF with the first-order Taylor expansion is used to cope with nonlinearities at the current time, while the UKF is concerned for the nonlinear function at the previous time. Using this hybrid extended-unscented Kalman filter, the accuracy of state estimation is improved since this allows for a third-order approximations for the nonlinear functions. A similar approach is taken for the design of the hybrid extended-cubature Kalman filter in [24]. The fractional-order average derivative method is used instead of the Grünwald–Letnikov difference method. The nonlinear functions are dealt with the extended Kalman filter (EKF) and cubature Kalman filter (CKF). The EKF with the first-order Taylor expansion is used to cope with nonlinearities at the current time, while the third-degree spherical-radical rule is used to produce cubature points for the functions in the state equation and output equation. The CKF uses this cubature points for effective state estimation in both uncorrelated and correlated noisy situations. Simulation results validate the effectiveness of the proposed approach. Additionally, fractional-order systems with non-Gaussian noises represent a hot topic in research regarding state estimation. The investigation of the FEKF is performed in [99] considering non-Gaussian white noises, such as Lévy noises [100]. A modified FKF algorithm is developed in [101] for discrete linear fractional-order systems under Lévy noises. An improved FKF is developed in [25] for discrete linear stochastic fractional-order system with measurement Lévy noise. The method is based on eliminating the maximum of the noise and then approximating the Lévy noise by a series of Gaussian white noises. Then, the principle of least squares is used to obtain the FKF. Two new Kalman filters for state estimation in fractional-order systems using colored measurement noise are developed in [102]. The methods are based on expanding measurement differencing method to produce new auxiliary outputs that turn the fractional-order system with colored measurement noise into a system with correlated process and measurement noises. The design of a discrete-time FKF is also developed in [103] for fractional-order systems with colored noises in the measured signals. The same problem is addressed in [11] where an FEKF is designed for nonlinear fractional-order systems perturbed by colored noises.

Several other extensions of the initial fractional-order Kalman filter have been developed, such as innovation-based fractional-order adaptive Kalman filter [16], the generalized fractional central difference Kalman filter [104] or a novel robust version [27]. The FEKF is also used for nonlinear discrete-time fractional-order systems using observations with multiple delays contaminated by additive white noise [99]. A new fractional singular Kalman filter is designed by [84] for the state estimation of discrete-time linear stochastic fractional-order singular systems using the deterministic least squares method. Later, the proposed approach is analyzed in terms of convergence and stability [105]. Numerical examples are provided to validate the results.

## 5. Applications of Fractional-Order Filters

The well-known Kalman filter is one of the most popular technique in the field of sensor fusion being employed to compensate the effect of sensor noise [106]. Applications of fractional-order filters cover mostly areas such as data transmission and networking issues, as well as estimations of state of charge in lithium-ion batteries (largely used in several industrial domains, including automotive industry). However, other applications of fractional-order filters cover areas such as aerodynamics, civil engineering, biomedical engineering, etc. This section covers some of the most recent research regarding applications of fractional-order filters.

### 5.1. Data Transmission and Networking

More practical problems occur when the physical data of a system are measured and analyzed through a network. Therefore, one of the practical areas are communication networks, where effort in analyzing the effect of packet losses has been highly considerable. To this kind of systems, generalization of Kalman filter algorithm can be applied [23,107,108]. For estimation of nonlinear systems, a set of generalized algorithms such as Extended Kalman filter (EKF) and Unscented Kalman filter are given in the literature [109,110,111]; especially, interesting algorithm is the Unscented Kalman filter that, in opposition to the Extended Kalman filter, not required differentiation of nonlinear function. In [110,112], UKF algorithm was used to teaching process of neural networks. In [87], the estimation results for fractional nonlinear systems based on Extended and Unscented Fractional Kalman filter (UFKF) were presented. This subsection offers a revision of some research papers dealing with communication networks.

A fractional-order transmitter in a noisy transmission channel is used in [113]. The transmitter is described as a fractional-order stochastic chaotic system. An extended fractional Kalman filter (EKF) is developed and employed as the received module and a synchronization scheme is designed to be used in cryptography in these systems. Different lemmas and theorems are presented in great detail, along with the proofs. Finally, the equations for the output of the communication channel are derived, which take into account the fact that the transmitter module might have another output that should be encrypted. The encryption/decryption methods are also presented. The proposed technique is tested via a fractional-order stochastic chaotic Chen system. The simulation results validate the theoretical part and show the effective performance of the proposed method in synchronizing fractional-order chaotic systems in the presence of noise.

Modified Kalman filters are also used in [87]. Here, due to the high nonlinearity of the processes involved an EFKF and an Unscented Fractional Kalman Filter (UFKF) are designed for online dual estimation algorithms for state variables and order estimation. Two situations are considered: direct and networked measurements. Different propositions about the filters are given and proved and then particularized for the proposed work. Several numerical examples are provided to validate the proposed approach. An analog circuit which represents in this case a fractional inertial system is also presented and used to test the developed estimation algorithm in three different conditions: direct measurements, networked measurements and networked measurements transmitted by real network. The conclusions suggest that the proposed technique is accurate enough and that it could be of great use in different applications involving estimation of real objects of unknown constant or variable order.

A modified fractional Kalman filter to reduce a common problem in network control systems, data packet dropouts, is presented in [114]. The authors argue that assuming an ideal information channel, without data dropouts, could be catastrophic. A weak network between the sensor and the FKF makes data dropout very likely with inaccurate information transmitted to the FKF. As a result, the whole filtering process is affected. The researchers provide for two possible solutions to this problem: the use of the last successfully transferred data packet which is simple, but very imprecise and inaccurate or the estimation of the missing packet according to the past results to compensate the missing data. A feasible implementation to the second solution is proposed, by introducing a new parameter γ that indicates how much information was transmitted successfully, along with the probability relationship between the measurement noise and γ. Then, the new parameter is introduced in the classical Kalman filter algorithm equations. The equations of the fractional Kalman filter (with the parameter γ) are obtained. The simulation results show that the estimations were fairly good, with a small error. The error between real and estimated value also tended to decrease as the probability of data dropout decreased.

A FKF solution is proposed for the problem of identifying malicious code and then, categorizing it according to its type (viruses, Trojans, spyware, etc.) [115]. A two-dimensional model commonly used in imagery is first used and adapted to a fractional-order state-space system representation. Then, the FKF design is presented including a priori estimation, variance, etc. Different methods of identifying the family of the malicious code are presented: image texturing, GIST feature extraction, etc. Simulation results are included that demonstrate that the new solution leads to better accuracy and robustness since it can ignore minor modifications of malicious code.

An improved fractional Kalman filter algorithm and its application to estimation problems over lossy networks is designed in [116]. The proposed algorithm improves not only the estimation process, but it also responsible for smoothing. The authors compare their proposed method with a fractional Kalman filter, and the numerical examples include the case when measured data are available directly from the plant. The simulation results show that significant improvements can be obtained using the proposed method, even if dropout problem in networks is important.

Medical, industrial, military fields use wireless sensors networks. Kalman filtering methods are used here to ensure accuracy and precision of sensor measurements. To estimate the states in sensors networks, a fractional-order distributed Kalman filter, as well as fractional diffusion Kalman filters are developed in [117]. A feasibility analysis is performed, with the simulations showing that the proposed algorithm leads to improved accuracy and efficiency compared to previous methods such as conventional fractional Kalman filter.

### 5.2. Applications Using Lithium-Ion Batteries

One of the applications of fractional-order filters is closely related to the field of electrical vehicles that employ lithium-ion batteries as their main energy source. The reliability of such batteries becomes of increasing importance. Batteries’ reliability depends heavily on their Battery Management System (BMS), which determines their State Of Charge (SoC) and State Of Health (SoH). SoC is a good indicator when it comes to mileage prediction, while SoH is a measure of the battery’s ability to store and deliver electrical energy. Efficient and non-destructive battery operation in automotive applications requires an accurate SoC estimation by the BMS [26]. As SoC cannot be measured by sensors, an estimation based on an equivalent circuit model of the lithium-ion battery is necessary. Traditionally, the equivalent circuit model consists of an integer-order model. For accurate simulation of the battery terminal voltage, the integer-order model needs a higher order, which causes a significant increase in the number of calculations. Apart from this, research on this topic has shown that many phenomena that occur in these batteries, such as mass transport [118] and the double-layer effect [119], can be well modeled by fractional-order calculus. At the same time, the fractional-order model uses less parameters to achieve higher accuracy [120]. In recent years, fractional-order calculus has been widely applied in battery modeling, from simplified models with fixed orders of differentiation [121] to more complex models with free differentiation orders [122,123,124]. A key drawback is that the order values are obtained using offline methods and do not adapt to changing conditions. A widely used method for estimating SoC based on the equivalent battery model consists of various extension of the Kalman filter.

To improve the BMS’ accuracy when it comes to SoH and SoC co-estimation, a fractional-order model is presented in [125]. First, the authors realize a fractional-order equivalent circuit model for the battery. Electrochemical impedance spectroscopy is used to measure the battery response to a multitude of frequencies. The results are used to determine a Nyquist plot that is later employed in the parameter identification procedure that uses global optimization algorithms such as Hybrid Genetic Algorithm and Particle Swarm Optimization (HGAPSO). Additionally, a dual fractional-order extended Kalman filter (DFOEKF) is designed for SoC and SoH estimation. The accuracy of the estimations using DFOEKF is also simulated with different tests. Finally, the battery is physically implemented, and final conclusions are drawn regarding the efficiency of the approach.

The estimation of SoC is also addressed in [126]. In practical implementations, the structure of a lithium-ion battery consists of multiple single battery cells that are connected (either in series or in parallel). To determine the state of each single battery cell, a BMS is employed in each lithium-ion battery. One of the most important parameters the BMS needs to determine is SoC. The authors of [126] propose a simple and feasible equivalent circuit model based on fractional variable-order approach. The estimation of SoC is done by an unscented fractional Kalman filter (UFKF). Its design is described in detail. First, some basic definitions of fractional-order derivatives are introduced, along with the equivalent model of the battery. Electrochemical impedance spectroscopy is used to measure the response of the lithium-ion batteries to different frequencies. A Nyquist plot can be derived based on the measured frequency response, as well as the physical circuit and the equations that describe the behavior of the lithium-ion batteries. These equations are later translated into a state-space model. Next, the equations for the sigma points generation, the state estimation time update, state error covariance time update, output update, state estimation measurement update and the state error covariance measurement update are formulated as well as the initialization of the filter. A dual filter is designed to address the problem of accuracy and quality of estimations. The necessary equations are reformulated, and a block diagram of the dual estimation is presented. Simulations of SoC estimations are then presented. The experimental setup is described and then the hybrid pulse power characterization test is conducted to acquire the offline parameters. After that, the federal urban dynamic schedule and Dynamic Stress Test (DST) are conducted to simulate real driving conditions. The results are promising as the proposed model can accurately describe the behavior of a lithium-ion battery and therefore can produce exact estimation of SoC. A fractional-order model combined with the fractional-order unscented Kalman filter is used in [127] to facilitate SoC estimation.

A study of SoC estimation under different ambient temperatures is performed in [128]. An equivalent circuit model of a lithium iron phosphate battery is established in the form of a first-order fractional model. Different charging and discharging battery capacity tests, as well as open circuit voltage tests were performed. The authors proposed a simplified modeling method considering hysteresis characteristics of open circuit voltage. The parameters of this model were identified at different temperatures based on a particle swarm optimization algorithm with dynamic inertia weight. Finally, the fractional extended Kalman filter was derived. Continuous Dynamic Stress Test conditions were used in the estimation of the battery SoC. The results showed that the estimation method had higher accuracy and increased robustness compared to the integer-order EKF.

Similar studies regarding different temperatures are presented in [26]. Here, both frequency domain information based on recorded impedance spectroscopy data and time domain information using a recursive least squares algorithm are used to derive a fractional-order model for lithium-ion batteries. The research provides for a straightforward and efficient way to identify the fractional orders based on recorded impedance spectroscopy. Then, an extended Kalman filter is designed to estimate the SoC. The results clearly show that the proposed approach using a fractional-order model and the designed fractional-order Kalman filter provides a higher accuracy and robustness compared to the classical method.

A cascaded fractional Kalman filter is designed in [129] as a solution for online estimation of SoC and the branch current in large battery structures. The approach is based on the fact that lithium-ion batteries are composed of multiple single cells. The model of the battery as well as the equivalent circuit model, equations and state space of a single cell are developed using fractional calculus. Battery branches are united using mesh currents as parameters. SoC is determined locally, which reduces the order of the computations. Only the total current needs to be measured, rather than each individual branch current. These are estimated using the cascaded fractional Kalman filter structure. The measurement setup is provided along with the results.

Several other papers offer different design procedures for fractional-order filters, usually in the form of extended version of the Kalman filter. An adaptive fractional-order extended Kalman filter is proposed in [130], for the estimation of state of energy (SOE). This index is also important for the electrochemical energy storage system in electric vehicles. The authors develop a physics-based fractional-order model with variable solid-state diffusivity to characterize the dynamic performance of a LiFePO${}_{4}$/graphite battery. The average current, as well as the average squared current is modeled since the available battery energy changes according to different applied load current profiles, the relationship between the remaining energy loss and the SoC. Different aging stages are considered, and the model parameters are updated automatically using a multi-step model parameter identification method based on the lexicographic optimization. An adaptive fractional-order extended Kalman filter is used to estimate the SOE with different operating conditions and different aging stages. The result presented demonstrate a small estimation error.

In [131], electrochemical impedance spectroscopy data are used to determine a fractional-order impedance model, to describe the polarization effect in a simple and meaningful way. Experimental data combined with genetic algorithms are used to identify the parameters of the model, as well as the fractional-order. To improve the computation efficiency, a fractional-order unscented Kalman filter technique is used, as well as the ‘short memory’ technique. The effectiveness of the proposed approach is demonstrated experimentally. The results show that show that the SoC estimation accuracy can be significantly improved using the proposed method, with an estimation error in the range of 3% [131]. An adaptive unscented particle filter for lithium-ion battery SoC based on an improved fractional-order model is also proposed in [132]. The algorithm uses the fractional orders as hidden parameters, which reduces the number of particles and hence the complexity of the algorithm iteration. A noise adaptive algorithm based on the residual sequence is employed, which solves the divergence problem of the filter and improves the adaptability. The experimental results show that in this case the SoC estimation is more accurate, the algorithm has strong robustness and fast convergence, and the evaluation index of the algorithm is the best, with a root mean-squared error of 0.67%. In [133], a fractional-order circuit model is used to predict battery dynamics. A new fractional-order model-based nonlinear estimator is proposed using a Luenberger term and a sliding mode term. Lyapunov’s direct method is used to design the estimator gains. Electric vehicle applications are used here as well. The proposed approach is validated, and comparative results are provided with other estimators to showcase the benefits of using the proposed method. The results demonstrate that the developed approach can estimate SoC with errors less than 0.03 in the presence of initial deviation and persistent noise. The Luenberger observer is also used for nonlinear fractional model-based SoC estimation in [124]. A direct Lyapunov method is used here to ensure the global asymptotic stability.

Fractional-order models are integrated with adaptive fractional-order EKF to estimate SoC, while updating part of the model parameters in [134,135]. A fractional-order EKF is also used in [136] to estimate battery SoC, where a fractional-order model with two Constant Phase Elements (CPE) is used to model the battery. Apart from using fractional-order filters in estimating battery state, these have also been used in SoC estimation for ultracapacitors. In [137], the results validate that the SoC estimator can precisely track the true SoC, and that the associated errors are less than around 2% in dynamic driving-cycle tests. The approach taken is very similar to that of SoC estimation in lithium-ion batteries. The SoC estimation is also tackled in [138] through an FOEKF filter. The study uses the Atangana–Baleanu fractional derivative to develop the fractional-order digital filter, which is further validated on a laboratory prototype with uplifting results.

### 5.3. Other Applications

Fractional Kalman filters and their more complex variants are also used in orientation problems in aerodynamics, in biomedical engineering or environmental issues, to name just a few. This subsection highlights some very recent applications of this kind.

Fractional-order complimentary filters are designed in [139] for small unmanned aerial vehicles to handle orientation. Most research papers use Kalman filters for this task and it produces good results when high-quality, high-cost sensors are used. However, in the case of low-cost, low-quality sensors, complementary filters are more adequate, since no assumptions are made with regards to linearity and noise statistics. The concepts of fractional calculus are extended to these types of filters and the results show that the proposed approach is indeed efficient on systems with non-Gaussian. In [106] a fractional Kalman filter (FKF) is implemented for attitude estimation of a moving vehicle. The input signals used are taken from a tri-axial MEMS (Microelectromechanical Systems) inertial sensors, i.e., accelerometer, magnetometer and gyroscope. Sensor fusion is performed on the measurements obtained by these sensors to obtain the vehicle’s roll, pitch and yaw angles. Sensor data captured from commercial navigation units is used in the FKF scheme. Reference attitude is used for comparative analysis. Several simulation case studies are performed that show that the estimation accuracy is highly dependent on system order. A robust central difference Kalman filter is designed in [27] and validated on the attitude determination system of a three-axis satellite including a star tracker and gyro sensor. The proposed approach is compared to numerous existing methods. The numerical simulation results for various case studies demonstrate the superior accuracy of the estimation method. A fractional-order gain Kalman filter is proposed in [17] for tracking vehicles by using fractional-order gain Kalman filter. To achieve this, the Kalman gain is modified using a feedback loop, which incorporates the fractional derivative of previous Kalman gains. The results show that the algorithm exhibits high accuracy for estimation of state-space variables, with the root mean square error improved by up to 17%. Robustness tests are also performed, with the overall conclusions that the proposed method demonstrates better capability than the standard Kalman filter.

Implicit and explicit approaches for fractional nonlinear model order estimation are covered in [28]. A benchmark model is used that links the applied angular rate to the neuron’s firing intensity within the vestibular system. As far as the implicit approach is concerned, several extended Kalman filters with fixed fractional-order nonlinear models running in parallel are used in an interacting multiple models scheme. The explicit approach is based on an augmented Unscented Kalman filter, where the fractional-order of the model is estimated explicitly within the filter state. Preliminary results on explicit joint state and model order estimation are presented. A sensor fusion scheme based on the fractional Kalman filter is presented in [140]. The Grünwald–Letnikov method is used to approximate the fractional-order terms in the FKF. Two different versions are developed and compared to integer-order conventional Kalman filters implementations. The case study considered here consists of a real-life limb tracking application. The filters are analyzed and the results are compared using a hand and a head motion data set, demonstrating the feasibility of the proposed approach. A biomedical application where fractional-order filters are used is presented in [68]. The study details the use of fractional-order filters to filter the myoelectric signal acquired from m. biceps brachii during isometric maximal voluntary contraction. Several ten test subjects are used to collect the data. The paper compares conventional and fractional Butterworth filters of two order groups in terms of offline filtration.

Air-quality is an important factor that needs to be monitored for several different reasons: it affects human life, climate change, meteorology, etc. In cases of decreased air-quality due to pollution, immediate measures should be taken into consideration. The aim of this paper [141] is to improve inverse air pollution emission and prediction in metropolitan areas. To achieve this, a chemical transport model coupled with the extended fractional Kalman filter (EFKF) is used. The EFKF is designed using a Matern covariance function and tuned by a genetic algorithm. The research covered in [141] argues that if the performance is affected by unknown disturbances or parameter variations, an EFKF may be a better option because fractional-order derivatives can be used for more accurate description of state variables. The technique is tested against measurement stations. Comparisons with a simple extended Kalman filter are also presented in terms of the mean-squared error. The results show that the EFKF gives a more accurate prediction due to its use of memorized results. A similar approach is also considered in [142]. The same chemical transport model as used in [141] is also considered in [143]. Instead of using an EFKF, the authors of Metia2016 propose a fractional Kalman filter. The results are compared with the standard Kalman filter using a root mean square error criteria. The conclusions drawn suggest that more accurate results can be achieved with a fractional Kalman filter than a simple Kalman filter. Additionally, the results demonstrate that the estimation becomes better when more iterations are done in the process. An extended fractional-order Kalman filter is also used in [144]. Indoor air pollution in smart buildings is the topic covered here, where an air-quality management system merging indoor air-quality index and humidex into an enhanced indoor air-quality index using sensor data on a real-time basis is proposed. Indoor air-quality index and humidex information are fused together using an FEKF with enhanced performance against measurement noise and nonlinearity, while indoor air pollutant levels are measured by a network of waspmote sensors. Based on the resulting enhanced indoor air-quality index, overall air-quality alerts are provided in a timely fashion. The method is validate using a case study.

An H-fractional extended Kalman filter algorithm is designed in [29] to estimate the stiffness and damping parameter of civil structures using noisy measurement data of the system response. Three load cases of engineering interest have been included in the research, including wind turbulences and wind induced waves in coastal engineering applications. In all cases, the accuracy of estimating the stiffness parameter was high. The damping parameter on the other hand was estimated only with satisfying accuracy. The method was compared to the standard extended Kalman filter (EKF). The results demonstrate that in the case of the EKF poor identification results for both the stiffness and the damping parameter were obtained, when neglecting the autocorrelation of the load process. An improved version of the fractional-order unscented Kalman filter is designed in [90] and applied to the electric pendulum model. Simulation results of this modified fuzzy FUKF algorithm show that the algorithm produces significantly better estimation results, especially when dealing with large initial estimation errors.

Image processing is another field that highly benefited from the advances of fractional-order filtering throughout the last decade. A review paper has been published in 2016 featuring the advances of fractional calculus in image filtering applications [145] showing that fractional-order filtering is a topic worth pursuing. The authors of [146] propose a fractional-order derivative filter to enhance image contrast using order prediction. The paper uses a Grünwald–Letnikov fractional-order mask where the fractional-order is determined in an adaptive manner, based on a prediction network built using a set of training images. Experimental results performed on multiple different images prove that fractional-order filters can be successfully used to improve the blur metric. Image denoising techniques have been successfully developed using fractional-order filters based on Alexander polynomials [147], Grünwald–Letnikov operator [148], total variations models [149] or Riemann–Liouville and Caputo models [150]. The authors of [151] propose a fractional-order principal component analysis theory and support vector machine algorithm for pattern recognition. The applicability of the study is proven in the biomedical field on highly similar digital images in ORL face databases. Several experiments are performed for both fractional-order and integer-order algorithms and it has been proven that the proposed fractional-order filtering method brings a 99.24% accuracy, significantly better than other eight comparison algorithms. Biomedical and image processing fields are also fused in [152] where a novel fractional-order filter is proposed for retinal blood vessel segmentation. Experimental data based on well-known biomedical databases prove the efficacy of the proposed algorithm as approximately 95%, showing a significant improvement than other methods. Another interesting application uses Grünwald–Letnikov to develop a fractional-order image filter for digital fingerprint identification [153].

## 6. Discussions

Fractional calculus in modeling and control applications has seen a rapid growth over recent decades. Several physical phenomena have been modeled using fractional calculus tools, while numerous research studies have shown that fractional-order controllers provide for better closed loop dynamics and robustness overall, but are definitely the suitable kind for controlling systems described by fractional-order models. Sensing and estimation is a crucial part for a closed loop system to work efficiently. It was then only a matter of time before several studies on fractional-order sensing and filtering methods emerged.

The purpose of this manuscript has been to gather a collection of the most important and relevant research papers covering fractional-order sensors, fractional-order analog and digital filters.

Section 3 shows that research on fractional-order sensors is rather limited at the moment. However, the references that have been mentioned in this paper show that more accurate data regarding the surrounding environment is possible to be collected using this type of sensing devices. The papers featuring the real-life construction of a fractional-order sensor have successfully proven the superiority of fractional-order measurements by improving the sensitivity and compensation of the nonlinear character of the transducer. The existing real-life applications are scarce, with a handful of papers featuring the experimental construction of a fractional-order sensor. These have been focused on measuring the quality of milk, the quality of drinking water and taste measurements in black tea liquor. The pattern suggests that quality of various fluids can be successfully assessed by impedance measurements using fractional-order sensors. The research trend shows that there is active work invested in building fractional-order sensors for physical phenomena such as pressure, force, displacement, currently only at a conceptual level, but with plenty of extension possibilities.

Fractional-order analog filters have emerged as consequences of the fractance device used for the development of fractional-order electronic components. However, current limitations in fractional-order physical hardware have led to scarce literature regarding the physical realization of fractional-order analog filters. The design of these filters is thoroughly studied and presented in a manifold of research works, mostly from a theoretic perspective. Most of these papers present the physical realization of the proposed analog filter from a conceptual perspective. However, the construction of analog fractional-order filters is limited by the need to create custom electronic components of fractional-order characteristics. The field of analog fractional-order filters will definitely benefit when fractional-order capacitors will be available commercially.

On the other hand, fractional-order digital filters are more abundant and various different approaches have been taken so far. By far, the most popular filtering techniques consist of fractional-order Kalman filters and various extensions such as the fractional-order extended Kalman filter and the fractional-order unscented Kalman filter. About 75% of the featured digital filtering papers focus on this topic, whereas the rest proposes variations of the Butterworth filter and fractional-order delay filters. The fractional-order Kalman filter, fractional-order extended Kalman filter, fractional-order unscented Kalman filter, robust extended fractional Kalman filter and fractional interpolatory cubature Kalman filters are used to deal with nonlinear fractional-order systems. Table 1 presents an overview of relevant papers associated with digital filtering of complex nonlinear systems.

Fractional-order Kalman filters and their extensions and improvements have been designed to deal with systems modeled by fractional-order equations. Kalman filtering via limited capacity or fading communication channels (networks) is a relevant problem raised by [154,155,156]. Reference [89], a very recent paper dealing with the issue of incomplete measurements and stochastic nonlinearities, addresses this topic through a state estimator based on robust fractional-order unscented Kalman filters. The results are compared with other types of filtering methods and the advantages of the proposed approach are highlighted. Similar conclusions are drawn based on the results obtained in [88], where both missing measurements and additive uncertainty in the gain are considered. Another problem related to Kalman filtering with irregular and/or intermittent measurements has been addressed in [157,158,159]. These aspects have been discussed by [91], another recent paper which clearly highlights the advantages of using a hybrid robust fractional interpolatory cubature Kalman filter instead of a traditional one.

As indicated above, the methods reviewed in the current manuscript are applied to handle similar problems as in [154,155,156,157,158,159]. The methods reviewed in this paper could be applied to the systems in [154,155,156,157,158,159], but the systems need to be altered and generalized to a fractional-order representation. This is not necessarily a problem, since it has been shown in several papers that fractional-order models can better represent the dynamics of natural phenomena. Hence, to use fractional-order filtering techniques for [154,155,156,157,158,159], first a fractional-order model of these systems must be estimated. Then, fractional-order filtering methods can be applied. Since fractional-order models capture better the significant dynamics of a system, compared to integer-order models, the conclusion would be that using this combination of fractional-order model+fractional-order Kalman filters would produce improved state estimation results compared to integer-order models and traditional filtering methods. A complete analysis and comparison between these approaches represents a viable research direction in the field of fractional-order filtering.

This paper has covered an extensive review of the most recent research papers dealing with fractional-order filters, both analog and digital. The last section of the paper provides for a survey of recent applications of these fractional-order filters. The main applicability has been associated with communication systems, followed by battery focused applications. The research trend associated with data transmission and networking applications is motivated by the need to filter physical data that is measured and analyzed through a network. Many relevant works prove the usage of the Kalman filter together with its variations to successfully compensate for the effect of packet losses and dropouts, malicious code identification, estimation problems in lossy networks and online estimation of state variables. Battery oriented applications are related to the automotive industry, especially in the field of electric cars manufacturing. Many phenomena associated with batteries can be accurately modeled using fractional-order derivatives, enabling fractional-order digital filters as a useful tool in estimating State of Charge (SoC) and State of Health (SoH). Proper estimation of both SoC and SoH are paramount for mileage prediction and the ability to store and deliver electricity. Other domains with real-life applications of fractional-order digital filters include filtering various signals related to aerial vehicles orientation, air-quality estimations, civil structure measurements. Furthermore, another interesting applicability consists of image processing, where fractional-order masks are applied to obtain different effects such as sharpening, denoising, etc. with multiple benefits in pattern recognition.

Despite the extensive reference list presented here, covering mostly research papers of the last decade, fractional calculus in sensing and filtering methods is still a topic that needs further research. Many problems need to be resolved, especially regarding the implementation of such methods on real processes. However, as Leibniz said in 1695 fractional calculus is a paradox that will someday lead to useful consequences. This review paper details how fractional calculus has made its mark on sensing and filtering methods with multiple useful consequences. Increasing research over the last period portrays a fractional calculus community on the brink of further expanding the useful consequences as prophetically envisaged by Leibniz.

## Figures and Tables

**Table 1:** Main fractional-order digital filter papers targeting nonlinear systems.

Title	Year	Reference
Extended and Unscented Filtering Algorithms in Nonlinear Fractional-Order Systems with Uncertain Observations	2012	[86]
Dual Estimation of Fractional Variable Order Based on the Unscented Fractional-Order Kalman Filter for Direct and Networked Measurements	2016	[87]
State-of-Charge Estimation for Lithium-Ion Batteries Based on a Nonlinear Fractional Model	2017	[124]
A Modified Fractional-Order Unscented Kalman Filter for Nonlinear Fractional-Order Systems	2018	[90]
A novel cubature statistically linearized Kalman filter for fractional-order nonlinear discrete-time stochastic systems	2018	[81]
Nonlinear Fractional-Order Estimator With Guaranteed Robustness and Stability for Lithium-Ion Batteries	2018	[133]
Robust extended fractional Kalman filter for nonlinear fractional system with missing measurements	2018	[88]
Fractional-order chaotic cryptography in colored noise environment using fractional-order interpolatory cubature Kalman filter	2019	[92]
Fractional-order Kalman filters for continuous-time linear and nonlinear fractional-order systems using Tustin generating function	2019	[78]
An adaptive unscented Kalman filter for a nonlinear fractional-order system with unknown order	2020	[98]
Design of a Robust State Estimator for a Discrete-Time Nonlinear Fractional-Order System With Incomplete Measurements and Stochastic Nonlinearities	2020	[89]
Extended Kalman Filters for Continuous-time Nonlinear Fractional-order Systems Involving Correlated and Uncorrelated Process and Measurement Noises	2020	[79]
Extended Kalman filters for nonlinear fractional-order systems perturbed by colored noises	2020	[11]
Hybrid extended-cubature Kalman filters for nonlinear continuous-time fractional-order systems involving uncorrelated and correlated noises using fractional-order average derivative	2020	[24]
Hybrid extended-unscented Kalman filters for continuous-time nonlinear fractional-order systems involving process and measurement noises	2020	[97]
Novel hybrid robust fractional interpolatory cubature Kalman filters	2020	[91]
Adaptive fractional-order Kalman filters for continuous- time nonlinear fractional-order systems with unknown parameters and fractional orders	2021	[96]
